# Multigenerational Study of Chemically Induced Cytotoxicity and Proliferation in Cultures of Human Proximal Tubular Cells

**DOI:** 10.3390/ijms151121348

**Published:** 2014-11-18

**Authors:** Lawrence H. Lash, David A. Putt, Bavneet Benipal

**Affiliations:** Department of Pharmacology, Wayne State University School of Medicine, 540 East Canfield Avenue, Detroit, MI 48201, USA; E-Mails: dputt5@yahoo.com (D.A.P.); bbenipal@mail.med.upenn.edu (B.B.)

**Keywords:** human kidney, proximal tubular cells, primary cell culture, apoptosis, proliferation, stress response

## Abstract

Primary cultures of human proximal tubular (hPT) cells are a useful experimental model to study transport, metabolism, cytotoxicity, and effects on gene expression of a diverse array of drugs and environmental chemicals because they are derived directly from the *in vivo* human kidney. To extend the model to investigate longer-term processes, primary cultures (P0) were passaged for up to four generations (P1–P4). hPT cells retained epithelial morphology and stained positively for cytokeratins through P4, although cell growth and proliferation successively slowed with each passage. Necrotic cell death due to the model oxidants *tert*-butyl hydroperoxide (tBH) and methyl vinyl ketone (MVK) increased with increasing passage number, whereas that due to the selective nephrotoxicant *S*-(1,2-dichlorovinyl)-l-cysteine (DCVC) was modest and did not change with passage number. Mitochondrial activity was lower in P2–P4 cells than in either P0 or P1 cells. P1 and P2 cells were most sensitive to DCVC-induced apoptosis. DCVC also increased cell proliferation most prominently in P1 and P2 cells. Modest differences with respect to passage number and response to DCVC exposure were observed in expression of three key proteins (Hsp27, GADD153, p53) involved in stress response. Hence, although there are some modest differences in function with passage, these results support the use of multiple generations of hPT cells as an experimental model.

## 1. Introduction

A large number of experimental models are available to study chemically induced nephrotoxicity (see [1] for recent review). These models include both laboratory animals (primarily rats and mice) for *in vivo* studies, and various *in vitro* models derived from kidneys of both laboratory animals and, less commonly, from humans. Most studies of chemically induced nephrotoxicity in the literature have been conducted with laboratory animals because of the ability to pair *in vivo* and *in vitro* experiments and the expense or lack of availability of human kidneys for *in vitro* studies. Some advantages exist in the use of rodent models. For example, the availability of transgenic mice, both knock-out and knock-in models, enables the study of specific proteins or processes in a controlled manner. It has long been recognized, however, that there are limitations in the use of laboratory animal models for predicting responses in humans due to both quantitative and qualitative species-dependent differences in metabolic and physiologic processes [2,3]. This is particularly true for the responses of the kidneys to many halogenated solvents, where male rats exhibit unique responses that do not occur in humans and make them highly susceptible to renal damage from such chemical exposures [4].

To address the potential problem of species differences and extrapolation from rodents to humans, primary cultures of human proximal tubular (hPT) cells have been developed as a model to study renal cellular function and responses to potentially toxic drugs and environmental chemicals [1]. Advantages include their reflection of *in vivo* biochemical properties and physiological function. Previous studies with this model have shown that the cells exhibit typical proximal tubular morphology [5,6,7,8], express a large array of both Phase I and Phase II drug metabolism enzymes [5,6,9,10], including cytochrome P450s, flavin-containing monooxygenases, UDP-glucuronosyltransferases, sulfotransferases, and glutathione *S*-transferases, and express and exhibit function of several major plasma membrane transporters for organic anions and cations [11], including the organic anion transporter 1 and 3, organic anion transporting polypeptide 1A2, P-glycoprotein, and several isoforms of the multidrug-resistance associated protein.

Besides validating transport and metabolic function, these cells have been used in the investigation of acute mechanisms of chemically induced cytotoxicity. Studies by both our laboratory [5,7,8,9,12,13,14,15] and others [16,17] have included exposures of hPT cells in suspension or in primary culture to the selective nephrotoxicant *S*-(1,2-dichlorovinyl)-l-cysteine (DCVC) or DCVC sulfoxide (which are the cysteine *S*-conjugate and cysteine *S*-conjugate sulfoxide metabolites, respectively, of the environmental contaminant trichloroethylene (TCE)), a variety of anti-inflammatory drugs, and inorganic mercury. Processes and responses investigated with this model have included necrotic and apoptotic cell death, cell proliferation and cell cycle alterations, mitochondrial dysfunction, and changes in gene expression.

While such acute cytotoxicity studies have provided useful, mechanistic information in an *in vitro* model derived directly from the human kidney, there is the limitation inherent with all such studies in primary cell cultures in that chemical exposures can only be conducted over a relatively limited time frame. Many types of cellular responses, such as those of interest in chemical carcinogenesis, require much longer exposure and assay times than are possible with primary cultures. In contrast to the use of primary cultures, which typically grow to confluence within five to nine days, a system capable of simulating exposures for weeks, or possibly longer, is needed. One option that many investigators have used has been immortalized cell lines. The only immortalized cell line derived from normal hPT cells is the HK-2 cell line [18]. Although many proximal tubular functions have been demonstrated to be retained by this cell line, the immortalization of the cells by viral transduction undoubtedly causes changes in cellular function, particularly with respect to stress response and proliferation capacity.

In the present study, we chose to circumvent the time-dependent limitations of primary cell culture by passaging primary cultures of hPT cells for multiple generations. At each generation, cytotoxicity responses of the cells to model toxicants and a well-characterized nephrotoxicant were studied by determining necrotic and apoptotic cell injury, cell cycle status, and proliferation. We further assessed cellular morphology, ATP content, redox defense, and expression of three key proteins involved in stress response. The results demonstrate that although hPT cells maintain their epithelial morphology, cellular energetics, and redox status, modest changes in sensitivity to toxicants are evident as cells are passaged for up to four generations. We conclude that passaging of primary cultures of hPT cells for up to four generations provides a reasonable model in which to study chemical exposures and cellular responses for up to several weeks. Additional studies are needed to more completely characterize hPT cell function during multiple generations of growth.

## 2. Results

### 2.1. Cellular Growth and Morphology

Primary cultures of hPT cells (designated as P0 cells) that are grown in serum-free, hormonally-defined media typically reach a state of near-confluence (80%–90%) in 5 to 7 days [6,7,8,10,11,12,13,14,15]. This typical pattern was observed in the present study. With successive passage, however, the rate of cell growth diminished. The decrease in growth rate was moderate during the first 2–3 passages (cells reached 80%–90% confluence in ~10, 12, and 15 days for P1, P2, and P3, respectively) but was considerably slower in P4 (cells reached 80%–90% confluence in 25–30 days).

**Figure 1:**
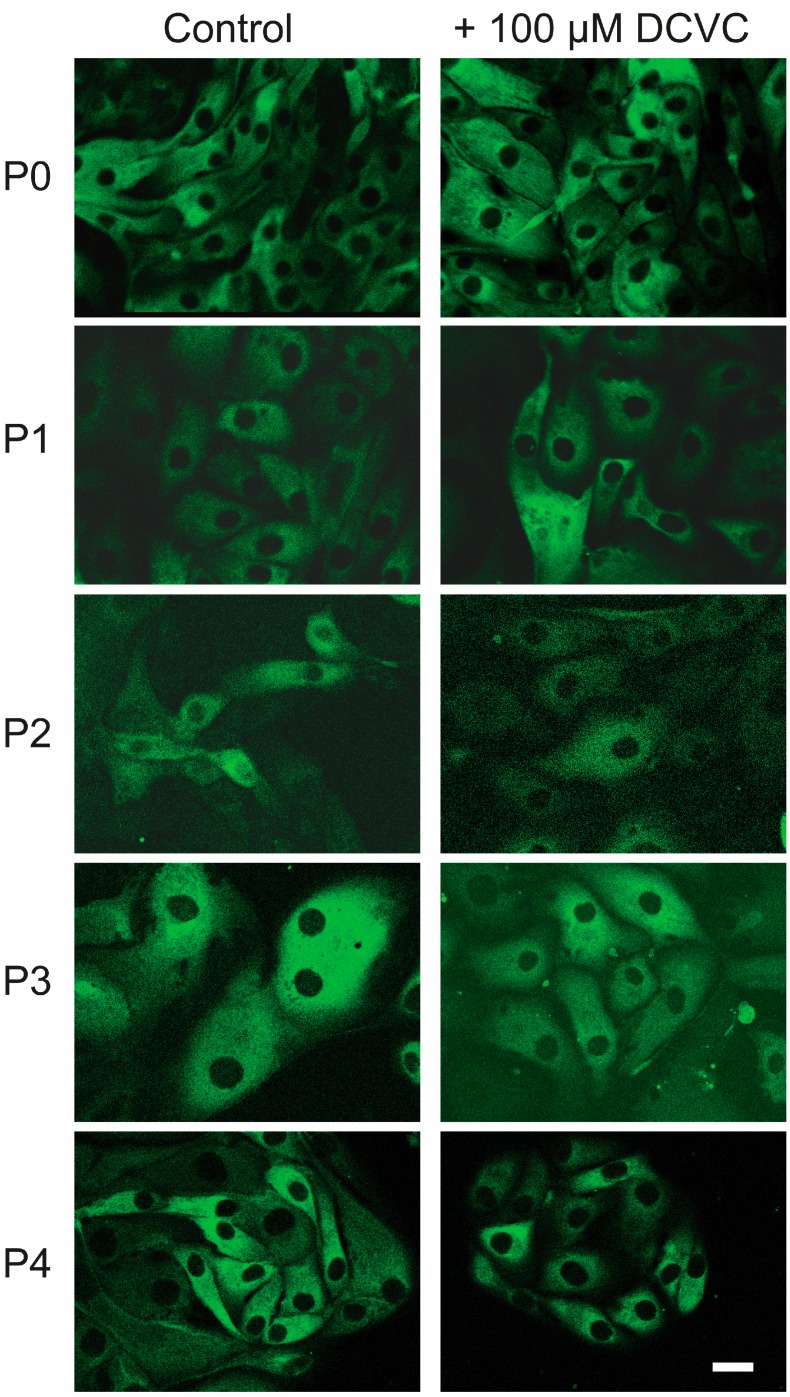
Cytokeratin staining in P0–P4 human proximal tubular (hPT) cells*.* hPT cells (approximately 80% confluent) at either primary culture stage (P0) or after one through four passages (P1–P4) were immunostained with a monoclonal anti-cytokeratin-fluorescein isothiocyanate (FITC)-conjugated antibody. Following 24-h incubations with either media or 100 µM *S*-(1,2-dichlorovinyl)-l-cysteine (DCVC), media was removed, cells fixed in a 3.7% (*v*/*v*) formaldehyde/phosphate-buffered saline (PBS) solution for 15 min, blocked with a 0.2% (*w*/*v*) bovine serum albumin solution for 45 min, and washed three times with a PBS solution containing 0.1% (*w*/*v*) saponin. The antibody was dissolved in the PBS/saponin solution and the cells were incubated overnight with the antibody. Cells were then washed with PBS and viewed under a confocal microscope for the presence of green fluorescence. Bar = 5 µm.

P0 hPT cells and those in P1 through P4 were incubated for 24 h with either cell culture medium or 100 µM DCVC. Epithelial morphology was assessed by immunofluorescent staining for cytokeratins and confocal microscopy (Figure 1). Under all conditions and from P0 through P4, cells stained positively for cytokeratins and exhibited typical epithelial morphology. No apparent differences were noted between control and DCVC-treated cells.

### 2.2. Comparative Cytotoxicity

**Figure 2:**
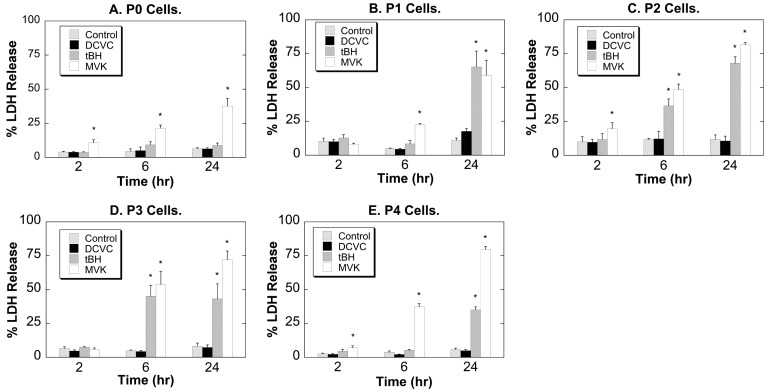
Chemically induced lactate dehydrogenase (LDH) release in P0–P4 hPT cells. hPT cells (approximately 80% confluent) at either primary culture stage (P0; **A**) or after one through four passages (P1–P4; **B**–**E**) were grown on collagen-coated 6-well plates and incubated for up to 24 h with either medium (=Control) or 100 µM DCVC, *tert*-butyl hydroperoxide (tBH), or methyl vinyl ketone (MVK). At the indicated times, LDH release was determined. Results are means ± SEM of measurements from 3 to 9 cell cultures. *, Significantly different (*p* < 0.05) from the corresponding control.

P0 through P4 hPT cells were incubated for 2, 6, or 24 h with either medium (Control) or 100 µM DCVC, *tert*-butyl hydroperoxide (tBH), or methyl vinyl ketone (MVK) and cell death was assessed by determination of LDH release (Figure 2). At the concentration tested (100 µM for each toxicant), the order of cytotoxic potency was generally MVK > tBH > DCVC. At all stages of cell culture and at either 2, 6, or 24 h at each passage, DCVC elicited little change in LDH release. In contrast, both tBH and MVK produced time-dependent increases in LDH release that were generally greater at the later passages. Thus, sensitivity to tBH and MVK increased with increasing passage number whereas cytotoxicity due to DCVC was modest and did not change with passage number.

**Figure 3:**
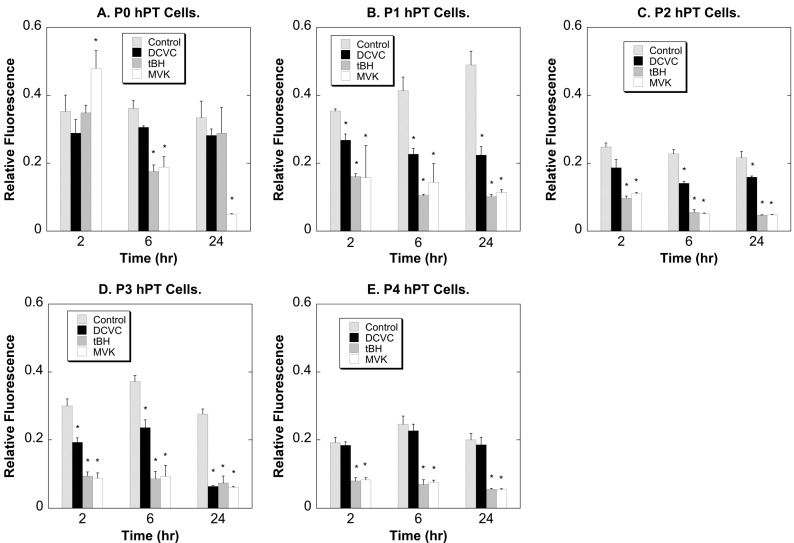
Chemically induced MTT reduction in P0–P4 hPT cells. hPT cells (approximately 80% confluent) at either primary culture stage (P0; **A**) or after one through four passages (P1–P4; **B**–**E**) were grown on collagen-coated 6-well plates and incubated for up to 24 h with either medium (=Control) or 100 µM DCVC, tBH, or MVK. At the indicated times, samples were incubated with MTT and fluorescence was read on a SpectraMax 2 plate reader. Results are means ± SEM of measurements from three separate cell cultures. *, Significantly different (*p* < 0.05) from the corresponding control.

To further assess the cytotoxicity of DCVC in hPT cells at different passage numbers, reduction of MTT was measured (Figure 3). MTT reduction requires mitochondrial activity and is generally considered an indicator of cell proliferation. The fluorescence of control cells, while remaining relatively constant over the 24-h incubation period during a given passage, tended to decrease after P1. Because MTT reduction occurs in mitochondria, this suggests that the density and/or activity of mitochondria in P2–P4 cells is lower than that in P0 or P1 cells. Based on changes in MTT reduction as an index of cytotoxicity or proliferation, 100 µM DCVC did not elicit any toxicity in P0 cells but was slightly toxic to P1–P3 cells. Both tBH and MVK produced a decrease in MTT reduction that was greater in P2–P4 cells than in either P0 or P1 cells.

Sensitivity to oxidative stress may be one factor that changes during the course of multiple passages. As an initial assessment of this hypothesis, activity of GPX was measured in P0 through P4 cells (Table 1). GPX activity modestly decreased over the course of four generations. However, the effect was modest, as activity in P4 cells was still 77% of that in P0 cells.

**Table 1:** GPX activity in P0–P4 hPT cells*.* Cell lysates of cultured cells grown on T-25 flasks were assayed for GPX activity by measuring NADPH oxidation at 340 nm. Results are means ± SEM of measurements from three separate cell cultures. *, Significantly different (*p* < 0.05) from the value in P0 cells.

Passage Number	GPX Activity (mU/mg Protein)
P0	60.4 ± 11.0
P1	37.9 ± 4.5 *
P2	50.0 ± 0.9
P3	59.6 ± 7.9
P4	46.3 ± 0.7 *

### 2.3. Effects of DCVC on Cell Cycle Distribution and Apoptosis

**Figure 4:**
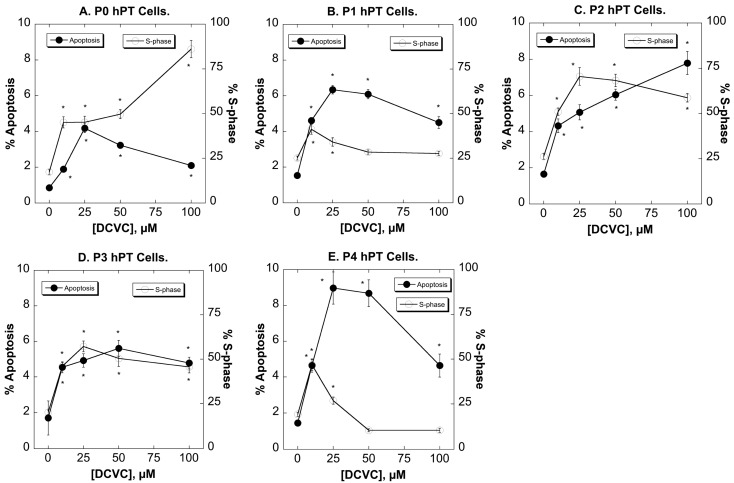
Effects of DCVC on cell cycle distribution and apoptosis in P0–P4 hPT cells*.* Cells (P0–P4; **A**–**E**) were grown on T-25 flasks until approximately 80% confluence in the presence of media (=Control) or from 10 to 100 µM DCVC. At the end of the incubation period, cells were removed from the matrix with an EDTA solution (Cellstripper™), cells were centrifuged at 400× *g*, collected, and treated with 1.0 mL of ice-cold 70% ethanol with vigorous mixing. Samples were frozen at −20 °C and then later processed for analysis by adding 0.5 mL of propidium iodide solution (50 µg/mL final) while mixing vigorously. The percentage of subdiploid cells (=apoptotic cells) and S-phase cells were determined by flow cytometry and FACS analysis. Results are means ± SEM of measurements from three separate cell cultures. *, Significantly different (*p* < 0.05) from the corresponding control.

DCVC induced apoptosis at concentrations of 10 to 100 µM, but this effect was generally modest and exhibited little difference in pattern in P0 through P3 cells (Figure 4). P4 cells, in contrast, exhibited slightly greater amounts of apoptosis due to DCVC, reaching a maximum at 25 µM DCVC that was approximately 50% higher than that in the earlier-passage cells. The pattern for the percentage of cells in S-phase as compared to DCVC concentration also did not differ significantly in P0 through P3 cells. However, P4 cells exhibited significantly less S-phase cells than earlier passage cells, particularly at DCVC concentrations ≥25 µM. This suggested that later generation cells, particularly those in P4, have a reduced capacity for proliferation. These data are also similar to those obtained with the MTT assay described above.

### 2.4. Effects of DCVC on Expression of Regulatory and Stress Response Proteins

**Figure 5:**
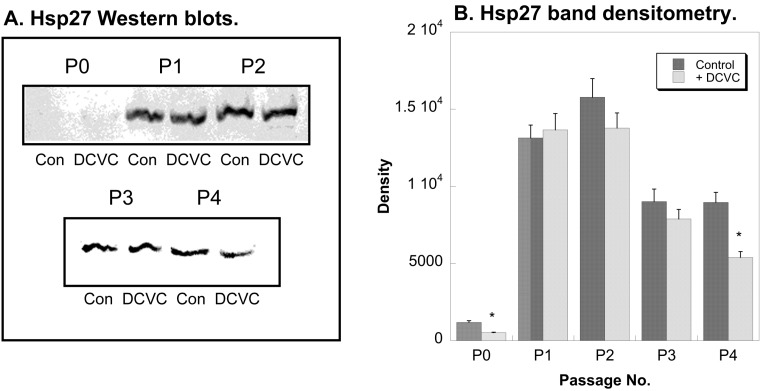
Effects of DCVC and passage number on Hsp27 expression in hPT cells*.* Cells (P0–P4) were grown on collagen-coated, T-25 flasks to approximately 80% confluence. Cells were incubated for 24 h with either media (Con = Control) or 100 µM DCVC. Protein expression was quantified by Western blot analysis with a mouse monoclonal antibody that recognizes human Hsp27, using either alkaline phosphatase staining and laser scanning densitometry (**A**) or enhanced chemiluminescence (ECL) (**B**). *, Significantly different (*p* < 0.05) from the corresponding control.

An important response of cells to chemical and environmental stresses is the induction of various proteins involved in the regulation of cell growth, death, and proliferation. Among these so-called stress response proteins, Hsp27 has been identified as an important cytoprotective protein in hPT cells [19,20]. Its induction leads to stabilization of the cytoskeleton and protection from various forms of cellular injury. In the present study, expression of Hsp27 protein in P0 through P4 cells exposed for 24 h to either media (=Control) or 100 µM DCVC was determined (Figure 5). Hsp27 expression was barely detectable in P0 cells, consistent with a lack of stress. DCVC had no significant effect on Hsp27 levels. In both Control and DCVC-treated cells, Hsp27 levels markedly increased in P1 and P2 cells but were slightly lower in P3 and P4 cells. Additionally, despite previous studies in P0 hPT cells showing induction of Hsp27 by DCVC [12], no significant differences were observed in the present study across generations of hPT cells.

**Figure 6:**
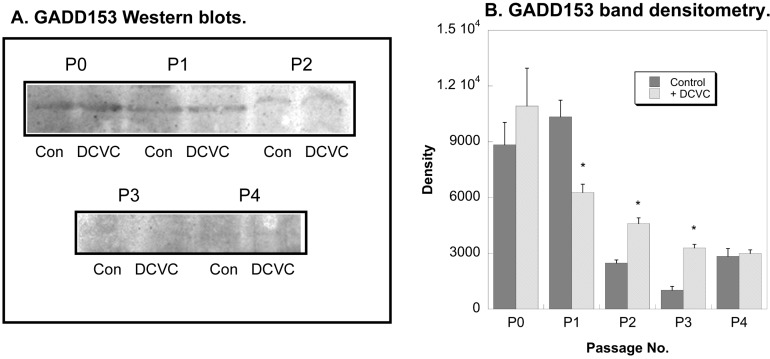
Effects of DCVC and passage number on GADD153 expression in hPT cells*.* Cells (P0–P4) were grown on collagen-coated, T-25 flasks to approximately 80% confluence. Cells were incubated for 24 h with either media (Con = Control) or 100 µM DCVC. Protein expression was quantified by Western blot analysis with a mouse monoclonal antibody raised against amino acids 1-168 of the full-length protein (*M*_r_ 30 kDa), using alkaline phosphatase staining (**A**) and laser-scanning densitometry (**B**). *, Significantly different (*p* < 0.05) from the corresponding control.

GADD153 is increased in PT cells in response to DNA damage from both DCVC and thiol oxidizing agents [21]. GADD153 protein levels were highest in P0 and P1 hPT cells but were markedly lower in P2 through P4 hPT cells (Figure 6), suggesting a diminished ability of those hPT cells to repair DNA damage with successive passage. DCVC modestly increased GADD153 protein levels only in P2 and P3 hPT cells.

**Figure 7:**
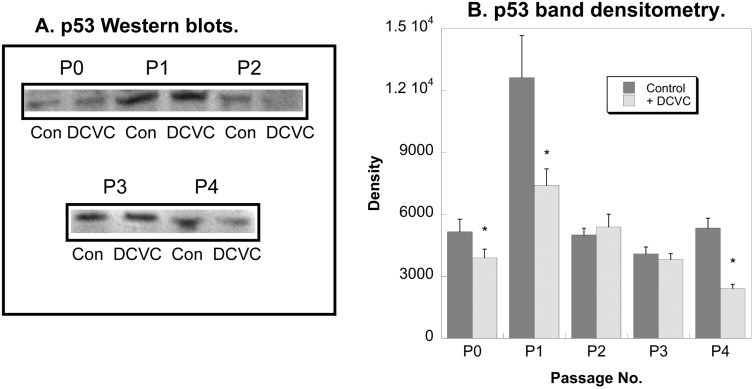
Effects of DCVC and passage number on p53 expression in hPT cells. Cells (P0–P4) were grown on collagen-coated, T-25 flasks to approximately 80% confluence. Cells were incubated for 24 h with either media (Con = Control) or 100 µM DCVC. Protein expression was quantified by Western blot analysis with a mouse monoclonal antibody raised against the *N*-terminus of human p53 (*M*_r_ 53 kDa), using alkaline phosphatase staining (**A**) and laser scanning densitometry (**B**). *, Significantly different (*p* < 0.05) from the corresponding control.

p53 is another cytoprotective protein that regulates cell growth, repair, and apoptosis in mammalian kidney in response to a variety of stresses [22,23,24]. p53 protein levels were readily detected and were generally maintained from P0 through P4 (Figure 7). Although previous studies in P0 hPT cells showed marked induction of p53 by DCVC [12], DCVC in the present study slightly decreased p53 levels in P0, P1, and P4 cells but had little effect in other generations.

## 3. Discussion

The present study was undertaken to provide a foundation for establishing passaging of primary cultures of hPT cells (P0 cells) as a model to study relatively long-term (*i.e.*, several days to weeks) responses to chemical and other stresses. In P0 cells, exposures can typically be conducted for up to 48 h [7,8,9,12,13,14,15]. However, responses such as changes in cell growth and proliferation and phenotype transformations that occur in response to carcinogens require an experimental model that can enable longer exposures. For passaged cells to be a viable model, however, several parameters and responses must be validated to ensure that conditions are relevant to the normal, *in vivo* PT cell. An alternative model would be an immortalized cell line. Although HK-2 cells are derived from human kidney and are commercially available [18], they are immortalized by viral transduction and do not completely respond like the *in vivo* hPT cell (Lash, L.H., and Putt, D.A., unpublished data).

Sens and colleagues [25,26,27,28,29,30,31,32,33] have published several studies with passaged hPT cells using second-generation hPT cells (equivalent to what we are calling P1 cells) to study heavy metal-induced toxicity (primarily cadmium and arsenic) and regulation of metallothionein gene expression. Several critical PT cell functions, including vectorial water transport and immediate-early gene expression in response to toxic chemical exposures, have been validated. These studies, however, have not addressed the issue of maintaining hPT cells through multiple generations and comparison of their properties and function to that of P0 hPT cells.

Although clear differences in growth rate and expression of some key regulatory proteins were observed with increasing passage number, several lines of evidence from the current study suggest that hPT cells, at least through the P2 stage, represent a viable *in vitro* model to study cellular and molecular responses of the human kidney to chemical and other stresses. Key lines of evidence included the maintenance of epithelial morphology, cytokeratin staining, and GPX activity through multiple generations. Cellular proliferation rate was within 50% of that of P0 cells through the P3 stage. Beyond that stage, however, it is clear that cellular properties and responses to chemical and environmental stresses will likely exhibit qualitative differences from what are observed in P0 cells. One potential limitation of the current study is that each hPT cell preparation was obtained from a different donor. Because individuals can exhibit variations in expression of various enzymes and membrane transporters [6,9,10,11], these individual differences may contribute to differences in proliferation and growth in each cell preparation. While this aspect has not been investigated in passaged cells, our previous experience has been that primary cultures of hPT cells from a large number of donors exhibit very similar growth rates [1,6,7,8,9,10,11,12,13,14,15]. Nonetheless, this is an important issue that requires further study.

An important feature of any *in vitro* toxicological model is that responses to well-characterized chemical toxicants are qualitatively and quantitatively similar to what are observed in the *in vivo* target cell. In the present work, we exposed P0 through P4 cells to both a specific nephrotoxicant (DCVC) and to two model toxicants that act by either oxidation or alkylation of low molecular weight thiols such as glutathione (tBH and MVK). While cellular responses to the two model toxicants were similar to what has previously been observed in primary cultures, an evident decrease in sensitivity to DCVC was observed in P3 and P4 hPT cells as compared to P0 hPT cells. Although there were some differences in experimental design between the present study and earlier studies on DCVC or DCVC sulfoxide cytotoxicity in P0 hPT cells [7,8,12,13,14], likely mechanisms to explain the changes in sensitivity include alterations in expression and/or function of membrane transporters and/or bioactivation enzymes and changes in mitochondrial function that occur with passage. The latter is critical because mitochondria are primary intracellular targets for the cytotoxic action of DCVC and reactive species that are derived from DCVC [8,14].

One potential limitation in the use of primary and passaged cultures of hPT cells is donor variability due to genetic polymorphisms in membrane transporters and drug metabolism enzymes, such as we have previously observed [1,6,9,10,11]. An alternative experimental model that is currently being developed and becoming more popular involves human stem cell-based approaches to generate proximal tubule-like cells [34,35,36,37,38]. Renal progenitor cells appear to play important roles in renal repair under various pathological conditions, such as repair of ischemia-reperfusion injury in mice [39,40,41]. Human induced pluripotent stem cells have been developed and used to demonstrate protection from ischemia-reperfusion and some forms of drug-induced injury [34,42]. Such cells have gained in popularity because of the safety and ethical concerns with the use of human embryonic stem cells. Further development of these models and validation of end points used to predict renal damage are still needed, but may lead to their more extensive application in the study of chemically induced nephrotoxicity.

Additional work is clearly needed to more fully characterize the energetic, drug metabolism, and transporter profiles of passaged hPT cells. With respect to energetic status, it is clear from the MTT assay results in the present study that mitochondrial function shows significant decreases from the P3 stage onward. Whereas glutathione *S*-transferase expression is well maintained during the course of both primary culture and through at least the P3 stage of passage [6], expression of so-called Phase I drug metabolism enzymes such as cytochrome P450s and flavin-containing monooxygenases are notoriously difficult to maintain, even in primary culture [6,10]. Further analysis of their expression during passage of hPT cells, with potential modifications in cell culture media or culture conditions may improve retention of their expression and activity. Expression and function of plasma membrane transporters for organic anions and cations are similarly difficult to maintain in culture [11]. hPT cells can adapt to culture conditions and changes in nutritional status by internalization of some plasma membrane transporters, making validation of their function and not just their expression levels critical.

In conclusion, the present study has investigated the utility of multiple generations of hPT cells as an experimental model for analysis of longer-term exposures and more complex processes than are possible in primary cultures of hPT cells (*i.e.*, P0 cells). Although additional parameters need to be studied and characterized, the present study has laid a foundation for use of hPT cells at least through the P2 generation as a valid model for toxicological studies.

## 4. Materials and Methods

### 4.1. Experimental Design

hPT cells were grown to approximately 80%–90% confluence on either collagen-coated 6-well plates, 24-well plates, or T-25 tissue culture flasks, depending on the parameter being measured. Supplemented cell culture medium (see below) was changed one day after plating or passage and then every other day thereafter. The concentration of toxicants used (100 µM) was based on previous studies in either rat proximal tubular (rPT) cells or hPT cells that elicited mild to moderate cell death by various assays.

### 4.2. Materials

TCE was purchased from Sigma Chemical Co. (St. Louis, MO, USA; Cat. No. T4928, ACS reagent, >99.5% purity). DCVC was synthesized from TCE and l-cysteine in sodium metal and liquid ammonia as described previously [43]. Purity (>95%) was assessed by high-performance liquid chromatography and thin layer chromatography and confirmed by ^1^H-NMR. All other chemicals for cell isolation and culture and for various assays were purchased from commercial vendors and were of the highest purity available.

### 4.3. Isolation and Primary Culture of hPT Cells

hPT cells were derived from whole human kidneys procured by the International Institute for the Advancement of Medicine (Edison, NJ, USA). All tissue was scored by a pathologist as normal (*i.e.*, derived from non-cancerous, non-diseased tissue). Cell isolation procedures were based on those originally described by Todd *et al.* [44] and modified [5,6] with use of sterile conditions (*i.e.*, all instruments and glassware were autoclaved and all buffers were filtered through a 0.2-μm pore-size filter). Renal cortex and outer stripe were cut into slices, washed with sterile phosphate-buffered saline (PBS), minced, and the pieces were placed in a trypsinization flask filled with 300 mL of sterile, filtered Hanks’ buffer, containing 25 mM NaHCO_3_, 25 mM HEPES, pH 7.4, 0.5 mM EGTA, 0.2% (*w*/*v*) bovine serum albumin, 50 μg/mL gentamicin, 1.3 mg/mL collagenase, and 0.59 mg/mL CaCl_2_, which was filtered prior to use. Whole kidneys were perfused with Wisconsin medium and kept on ice until they arrived at the laboratory, which was usually within 24 h of removal from the donor. Kidneys used for these studies came from 14 donors (age, mean ± SEM): 8 males (48.1 ± 4.5), 6 females (56.3 ± 2.5).

All buffers were continuously bubbled with 95% O_2_/5% CO_2_ and were maintained at 37 °C. Minced cortical pieces from whole kidneys were subjected to collagenase digestion for 60 min, after which the supernatant was filtered through a 70-μm mesh filter to remove tissue fragments, centrifuged at 150× *g* for 7 min, and the pellet resuspended in Dulbecco’s Modified Eagle’s Medium:Ham’s F12 Medium (DMEM:F12; 1:1). Approximately 5 to 7 × 10^6^ cells were obtained per 1 g of human kidney cortical tissue. No further purification beyond separation of cortical tissue and filtration through mesh was conducted with the hPT cells. Previous studies [5,6] indicated that the hPT cells isolated in this manner are >80% of proximal tubular origin.

hPT cells were resuspended in 2 mL of DMEM:F12 and diluted to 500 mL with cell culture medium, which was serum-free and hormonally-defined. Composition of this supplemented medium was based on earlier work establishing optimal conditions for primary culture of rat PT cells [19,45]. Basal medium was a 1:1 mixture of DMEM:F12. Standard supplementation included 15 mM HEPES, pH 7.4, 20 mM NaHCO_3_, antibiotics for day 0 through day 3 only (192 IU penicillin G/mL + 200 μg streptomycin sulfate/mL or 50 µg gentamicin/mL) to inhibit bacterial growth, 2.5 μg amphotericin B/mL to inhibit fungal growth, 5 μg bovine insulin/mL (=0.87 µM), 5 μg human transferrin/mL (=66 nM), 30 nM sodium selenite, 100 ng hydrocortisone/mL (=0.28 µM), 100 ng epidermal growth factor/mL (=17 nM), and 7.5 pg 3,3',5-triiodo-dl-thyronine/mL (=111 nM). Cells were seeded at densities of 50–100 μg protein per cm^2^ (0.5 × 10^6^–1.0 × 10^6^ cells/mL) on either 6-well plates, 24-well plates or polystyrene T-25 tissue culture flasks, depending on the needs of the experiment. Volumes were 0.5 mL per well for 6- and 24-well plates or 1.5 mL for T-25 flasks. Cultures were grown at 37 °C in a humidified incubator under an atmosphere of 95% air/5% CO_2_ at pH 7.4. Cultures were grown to approximately 80%–90% confluence (generally 5–6 days for primary cultures [P0 cells]) prior to experiments, unless otherwise stated. Cells were harvested by either scraping the flasks with a Teflon scraper or by brief incubation with Cellstripper (Cellgro, Herndon, VA, USA) (in Ca^2+^- and Mg^2+^-free Hanks’ buffer).

### 4.4. Passaging of hPT Cells

For passage, cells were grown on collagen-coated T-25 flasks and briefly incubated with Cellstripper in Ca^2+^- and Mg^2+^-free Hanks’ buffer. Cells were re-seeded at densities of 50–100 μg protein per cm^2^ (0.5 × 10^6^–1.0 × 10^6^ cells/mL) on collagen-coated T-25 flasks for subsequent passaging (up to P4) or on 6- or 24-well plates, depending on the needs of the experiment, as described above. For subsequent passage, cells were grown to 80%–90% confluence prior to passage.

### 4.5. Immunocytochemical Staining for Cytokeratins

Cytokeratins were monitored as an epithelial cell marker of hPT cells on Day-5 of culture at each passage. Following fixation with 3.7% (*v*/*v*) formaldehyde and blocking with 0.2% (*w*/*v*) bovine serum albumin, cells were incubated with a monoclonal anti-pan cytokeratin antibody conjugated to fluorescein isothiocyanate (FITC) (Sigma Chemical Co.) in PBS containing 0.1% (*v*/*v*) saponin. The stained cells were viewed and photographed with a Zeiss LSM 310 confocal laser-scanning microscope.

### 4.6. Measurement of Cell Death by Lactate Dehydrogenase (LDH) Release

Cell death due to either necrosis or late-phase apoptosis was determined by measurement of LDH release from cells after incubation with the indicated toxicants for 2, 6, or 24 h. LDH activity was determined in media and, after removal of media, washing of cells with PBS, and solubilization of cells with 0.1% (*v*/*v*) Triton X-100, in total cells, by addition of pyruvate and NADH and following the decrease in A_340_. The fraction of LDH release was calculated by the formula: % LDH release = [LDH activity in media/(LDH activity in media + LDH activity in total cells)] × 100%. Based on previous studies of chemically induced cell death in primary cultures of hPT cells [7,8], LDH release at the 2- and 6-h time points is likely due primarily to necrosis whereas that at the 24-h time point is likely due to both necrosis and apoptosis.

### 4.7. Measurement of Cytotoxicity and Proliferation by MTT Assay

Cytotoxicity and cell proliferation were measured with the MTT (3-(4,5-dimethylthiazol-2-yl)-2,5-diphenyltetrazolium bromide) Cell Proliferation Assay kit (ATCC, Manassas, VA, USA) and was expressed in treated samples as the percent of control measurements. Cells were grown on collagen-coated, 24-well plates and incubated with the indicated concentration of toxicant for 2, 6, or 24 h. At the end of the incubation period, media were replaced with fresh media and the MTT reagent was added to each well. Cells were periodically viewed under a phase contrast microscope for the appearance of the intracellular punctate purple precipitate. Once the precipitate was clearly visible, detergent was added to lyse cells and the plate was incubated in the dark for at least 2 h, after which time the absorbance at 570 nm was measured in a SpectraMax2 plate reader (Molecular Devices, Sunnyvale, CA, USA).

### 4.8. Flow Cytometry Assay of Cell Cycle Distribution and Apoptosis

Cell cultures were washed twice with sample buffer (PBS plus glucose (1 g/L) filtered through a 0.22-μm filter), dislodged by trypsin/EDTA (0.1% *w*/*v*) incubation, centrifuged at 400× *g* for 10 min, and resuspended in sample buffer. Cell concentrations were adjusted to 1 to 3 × 10^6^ cells/mL with sample buffer and 1 mL of cell suspension was centrifuged at 400× *g* for 10 min. All of the supernatant except 0.1 mL/10^6^ cells was removed and the remaining cells were mixed on a vortex mixer in the remaining fluid for 10 s. Next, 1 mL of ice-cold ethanol (70%, *v*/*v*) was added to the sample drop by drop, with samples being mixed for 10 s between drops. The tubes were capped and fixed in ethanol at 4 °C. After fixation, cells were stained in propidium iodide (50 μg/mL) containing RNase A (100 U/mL). Samples were then mixed, centrifuged at 1000× *g* for 5 min and all the ethanol except 0.1 mL was removed. Cells were mixed in the residual ethanol and 1 mL of the propidium iodide staining solution was added to each tube. After mixing again, cells were incubated at room temperature for at least 30 min. Samples were analyzed within 24 h by flow cytometry using a Becton Dickinson FACSC*alibur* Flow Cytometer, which is part of the Microscopy, Imaging and Cytometry Resources Core at Wayne State University. Analysis was performed with 20,000 events per sample using the ModFit LT version 2 for Macintosh data acquisition software package (Verity Software House, Inc., Topsham, ME, USA; distributed by Becton Dickinson Immunocytometry System (BDIS), San Jose, CA, USA). Propidium iodide was detected by the FL-2 photomultiplier tube. Fractions of apoptotic cells were quantified by analysis of the sub-G_1_ (sub-diploid) peak with ModFit cell cycle analysis. The percent distribution of cells in the various stages of the cell cycle (G_0_/G_1_, S, G_2_/M) was also calculated. Cell aggregates were discarded in the flow cytometry analysis by post-fixation aggregate discrimination.

### 4.9. Western Blot Analyses

Total cellular protein (150 µg) was loaded in wells of 10% polyacrylamide gels. After electroblotting of protein onto nitrocellulose paper, blots were blocked for 1 h in 5% milk powder solution and incubated overnight with the primary antibody to heat shock protein 27 (Hsp27), growth and DNA damage protein153 (GADD153), or p53. The antibody for Hsp27 was a mouse monoclonal antibody that recognizes human and monkey Hsp27 (*M*_r_ 27 kDa) (StressGen, Victoria, BC, Canada). The antibody for GADD153 was a mouse monoclonal antibody raised against amino acids 1–168 of the full-length mouse GADD153 (*M*_r_ 30 kDa) (Santa Cruz Biotechnology, Santa Cruz, CA, USA). The antibody for p53 was a mouse monoclonal antibody raised against the *N*-terminus of human p53 (*M*_r_ 55 kDa) (Cell Signaling, Beverly, MA, USA). Blots were then washed three times with Tris-buffered saline containing Tween-20 (TTBS) and incubated with a secondary antibody conjugated to alkaline phosphatase (Jackson ImmunoResearch, West Grove, PA, USA) for 1 h. Blots were washed 3–6 times and then assayed for color development using BCIP/NBT as substrates (Promega, Madison, WI, USA). Alkaline phosphatase staining intensity was determined by scanning laser densitometry with a Fuji Science Imaging System (Fuji Health Science, Burlington Township, NJ, USA) connected to a Macintosh G4 computer.

### 4.10. Data Analysis

Where appropriate, values were normalized to cellular protein content. Protein was measured using the bicinchoninic acid protein assay kit (Pierce, Rockford, IL, USA) and measuring absorbance at 532 nm. Standard curves using bovine serum albumin as the standard were generated to determine sample protein concentrations. Significant differences between mean values of controls and treated samples were first assessed by a one-way or two-way analysis of variance. When significant *F* values were obtained with the analysis of variance, the Fisher’s protected least-significance *t* test was performed to determine which means were significantly different from one another, with two-tail probabilities <0.05 considered significant.

## Abbreviations

DCVC: *S*-(1,2-dichlorovinyl)-l-cysteine
DMEM:F12: Dulbecco’s Modified Eagle’s Medium:Ham’s F12 Medium
FITC: fluorescein isothiocyanate
GADD153: growth and DNA damage protein153
GPX: GSH peroxidase
GSH: glutathione
hPT: human proximal tubular
Hsp27: heat shock protein 27
LDH: lactate dehydrogenase
MTT: 3-(4,5-dimethylthiazol-2-yl)-2,5-diphenyltetrazolium bromide
MVK: methyl vinyl ketone
P0: primary cell cultures
P1–P4: cells passaged one to four generations
PBS: phosphate-buffered saline
rPT: rat proximal tubular
tBH: *tert*-butyl hydroperoxide
TCE: trichloroethylene
TTBS: tris-buffered saline containing Tween-20
